# An antisense antidote to oncogenic poison exons

**DOI:** 10.1101/gad.353988.126

**Published:** 2026-09-01

**Authors:** René M. Arvola, Guramrit Singh

**Affiliations:** Department of Molecular Genetics, Center for RNA Biology, The Ohio State University, Columbus, Ohio 43210, USA

**Keywords:** antisense oligonucleotide therapy, cancer, gene expression, nonsense-mediated mRNA decay, RNA splicing

## Abstract

Splicing factors are frequently mutated in myeloid cancers, causing splicing aberrations that derail the expression of tumor suppressor genes. In SRSF2 mutated cancers, a key oncogenic splicing event is the inclusion of a “poison” exon that introduces an early stop codon in *EZH2* mRNA, causing its destabilization. In this issue of *Genes & Development*, Islam et al. (doi:10.1101/gad.353628.126) define how mutant SRSF2 binding to the poison exon mediates its inclusion and identify an antisense oligonucleotide that represses the exon to restore EZH2 function and rescues hematopoietic defects. Thus, targeting of poison exons, many of which show protumorigenic and antitumorigenic properties, is a promising new avenue to treat cancer.

## Abstract (precis)

In this Outlook, Arvola and Singh discuss a study in this issue of *Genes & Development* by Islam et al. that identifies and targets a poison exon inclusion event that controls EZH2 expression in SRSF2 mutated cancer. The authors highlight recent advances in targeting such poison exons with antisense oligonucleotides for new cancer therapeutics.

Cancer cells exploit a variety of mechanisms, including pre-mRNA splicing, to outpace cellular checks and balances to grow, proliferate, and spread. RNA splicing dysregulation is a hallmark of tumor cells. Striking examples of oncogenic RNA splicing changes are caused by recurrent mutations in splicing factor genes (e.g., *SRSF2* and *SF3B1*) in cancers of myeloid lineage (Anczukow et al. 2024). Widespread changes in RNA splicing patterns in these malignancies are conventionally thought to cause ripple effects on gene expression by altering protein isoforms. In this issue of *Genes & Development*, Islam et al. (2026) showcase another kind of oncogenic splicing aberration in *SRSF2* mutated myeloid cancer cells that “poisons” protein coding capacity of a tumor suppressor gene via inclusion of a highly conserved exon that contains a premature termination codon (PTC). By showing that antisense oligonucleotide (ASO)-mediated reversal of this oncogenic splicing event corrects the gene function and consequential hematopoietic defects in leukemic cell lines, they make a case for considering splice alterations beyond protein isoform switching in cancer.

Among the splicing factors mutated in myelodysplastic syndromes (MDSs), SRSF2 is unique, as it primarily binds to short exonic splicing enhancer (ESE) sequences to promote exon inclusion by promoting 5′ and 3′ splice site recognition. MDS-driving mutations in SRSF2 at a singular hotspot, proline 95 (to histidine, leucine, or arginine; SRSF2^Mut^), alter its RNA binding specificity from G-rich (GGWG) to C-rich (CCWG) motifs (Kim et al. 2015; Rahman et al. 2020). Islam et al. (2026) first confirmed a previous observation that in leukemic patient samples, a prominent consequence of altered RNA recognition by SRSF2^Mut^ is inclusion of “poison exons” that introduce a PTC to disrupt open reading frames of host genes and trigger nonsense-mediated mRNA decay (NMD) (McGlincy and Smith 2008). Poison exons are often embedded within ultraconserved regions in mammalian genomes and are best understood as autoregulatory feedback modules for the SR protein family and other splicing factors (McGlincy and Smith 2008; Leclair et al. 2020). Recently, many of these highly conserved yet poorly investigated genetic modules were shown to act as tumor suppressors by attenuating expression of genes with oncogenic functions (Thomas et al. 2020). Thus, poison exons have an untapped potential in understanding cancer progression and devising new therapies.

Taking cues from previous studies, Islam et al. (2026) home in on a poison exon within the *EZH2* gene that is exclusively included in SRSF2^Mut^ patients, leading to its downregulation via NMD. *EZH2* encodes the methyltransferase subunit of polycomb repressive complex 2 (PRC2) that silences genes through H3K27 trimethylation. As EZH2/PRC2 targets genes involved in cell fate decisions, it plays a tumor-suppressive role as a regulator of hematopoiesis, and mutations within the *EZH2* gene itself are linked to myeloid cancers (Ernst et al. 2010). Thus, *EZH2* is a prime target in SRSF2^Mut^-driven cancers.

To test the hypothesis that *EZH2* poison exon inclusion is the key splicing change that drives leukemogenesis due to altered binding specificity of SRSF2^Mut^, Islam et al. (2026) next pinpoint the precise RNA recognition events that drive poison exon inclusion. They identify three CCWG motifs within the *EZH2* poison exon as potential mediators of the novel inclusion event. Of these motifs, the first site is the key element necessary for SRSF2^Mut^-dependent poison exon inclusion (Fig. 1A). Moreover, artificially tethering wild-type SRSF2 or SRSF2^Mut^ at the first motif site is sufficient to drive poison exon inclusion. Notably, SRSF2^Mut^ binding to the poison exon also enhances recruitment of NMD-promoting factors to *EZH2* mRNA, thus ensuring its strong downregulation.

**Figure 1.**
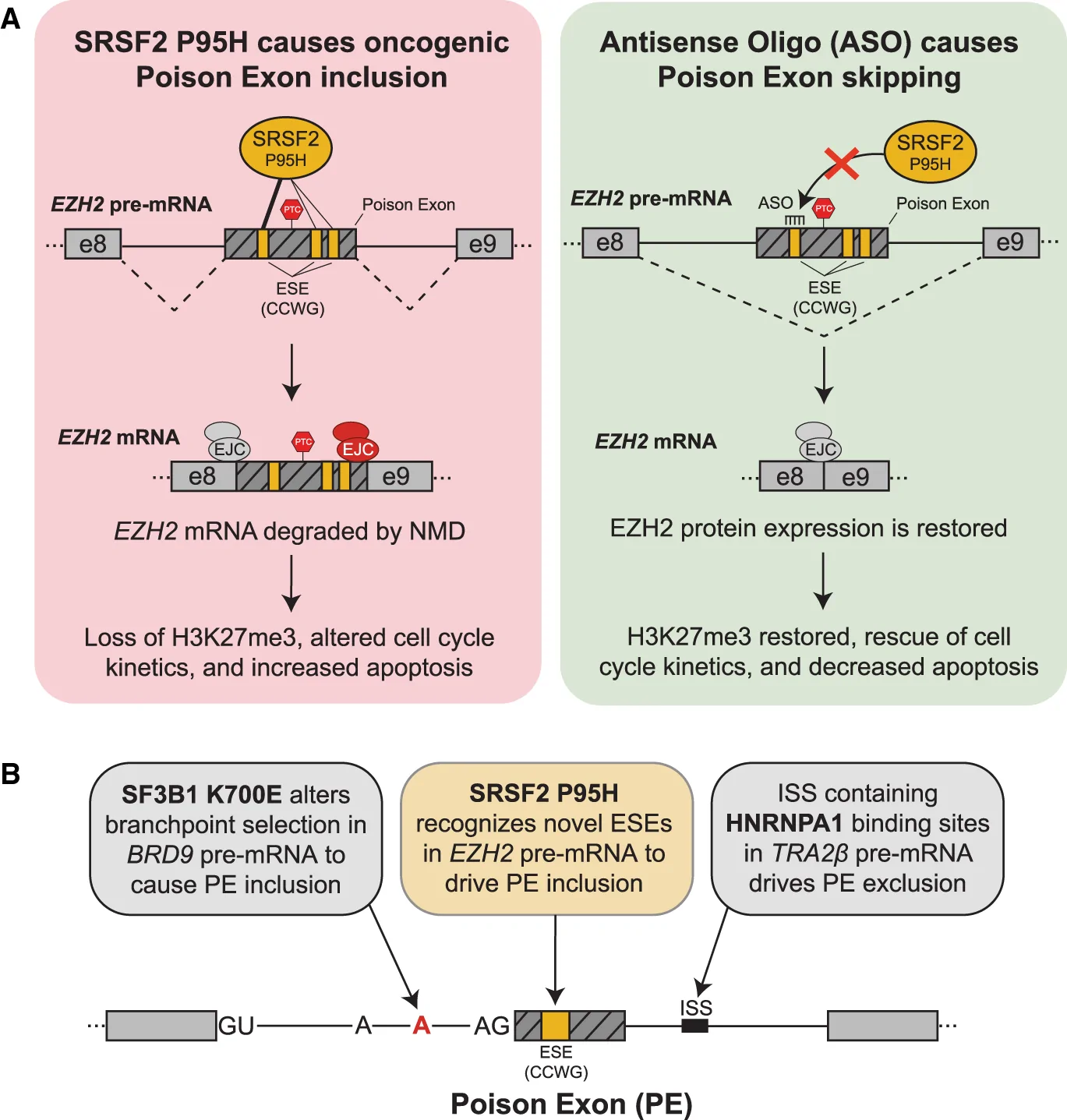
Poison exon usage and modulation in cancer. (*A*) SRSF2 P95H drives an oncogenic poison exon inclusion event that can be corrected with ASO targeting. (*Left*) Of the three novel exon splicing enhancer (ESE; yellow bars) sequences within the *EZH2* poison exon (rectangle with oblique lines), the SRSF2 P95H mutant binding to the first site (indicated by a thicker line connecting SRSF2 P95H to ESE) leads to poison exon inclusion, introducing a premature termination codon (PTC; red shape) and increasing the deposition of the exon junction complex (EJC) and NMD factors (red and gray ovals). The resulting *EZH2* transcript is degraded by NMD, and this downregulation of EZH2 leads to loss of its activity as a histone methyltransferase and tumor suppressor that silences genes involved in cell fate decisions. (*Right*) Islam et al. (2026) show that an ASO blocking access to the first ESE bound by the mutant SRSF2 leads to skipping of the poison exon and restoration of EZH2 expression and cellular functions. (*B*) Splicing factors and *cis* elements that drive the oncogenic inclusion or exclusion of poison exons. (*Left*) SF3B1 mutants alter branchpoint selection by recognizing an adenosine aberrantly proximal (red A) to the 3′ splice site in *BRD9* pre-mRNA. (*Middle*) In this issue of *Genes & Development*, Islam et al. (2026) define novel ESEs that lead to poison exon inclusion in *EZH2* pre-mRNA. (*Right*) An intronic splicing silencer (ISS; thick black line) with HNRNPA1 binding sites drives poison exon exclusion in the *TRA2*β oncogene.

With knowledge in hand about the precise SRSF2^Mut^ binding event that drives *EZH2* downregulation, the investigators turn to ASOs to interfere with this oncogenic splicing event. ASOs are short synthetic nucleic acid sequences complementary to RNA regions of interest that can occlude recognition sites of splicing factors to cause splice switching. Indeed, an ASO targeting an intronic splicing silencer within the *SMN2* gene promotes inclusion of its exon 7 to boost production of functional SMN protein and is in clinic to treat spinal muscular atrophy (SMA) (Bennett et al. 2019). Of the four ASOs tested by Islam et al. (2026), ASO2 that overlaps with the CCWG motif at the key first site efficiently represses *EZH2* poison exon inclusion (Fig. 1A). Consequent elevation of EZH2 protein levels and activity in leukemic cell lines increases H3K27 trimethylation, rescues cell cycle perturbations, and decreases apoptosis.

This work bolsters the emerging paradigm that poison exons are an effective target to combat oncogenesis (Fig. 1B). A proof of concept was first provided in the case of MDS-causing mutations in SF3B1 that alter its intron branchpoint recognition properties. Poison exon inclusion in *BRD9* mRNA and consequential downregulation of BRD9 are critical oncogenic events driven by aberrant branchpoint recognition by mutant SF3B1. By blocking the aberrant branchpoint sequence using an antisense morpholino, analogous to an ASO, BRD9 expression and its tumor-suppressive functions as a chromatin remodeler are restored, ultimately slowing tumor growth in mouse melanoma xenografts (Inoue et al. 2019). A converse approach using ASOs to promote poison exon inclusion has also been described to combat tumorigenesis. In breast cancer, a poison exon in *TRA2*β gene is skipped more often, leading to upregulation of this SR protein and in turn causing downstream splicing changes. After systematically defining an HRNPA1-regulated intronic splicing silencer as a determinant of *TRA2*β poison exon exclusion, Leclair et al. (2020) used an ASO targeting this site to downregulate *TRA2*β expression by promoting poison exon inclusion, thereby slowing breast cancer cell growth in culture. Considering that 61 poison exons including those in *SR* and *hnRNP* splicing factor genes show antitumorigenic properties, whereas another subset can promote tumorigenesis (Thomas et al. 2020), this class of conserved exons could very well serve as a “hit list” of candidates for splice event-specific targeted cancer therapeutics.

In summary, the work by Islam et al. (2026) highlights the recent success of preclinical studies to target poison exons using ASOs for anticancer therapies. The breakthrough of splice-switching ASOs to treat SMA has brightened the outlook for antisense therapies for cancer. Indeed, many anticancer ASOs, though primarily geared toward protein isoform switching, are under intense preclinical testing (Anczukow et al. 2024). However, challenges such as ASO delivery and penetrance, as well as insufficiency of single-gene (or exon) targeting, will have to be surmounted to drive these promising avenues to the clinic. Nonetheless, poison exons have emerged as yet another Achillies’ heel that can be exploited to combat cancer cells.

## References

[GAD353988ARVC1] Anczukow O, Allain FH-T, Angarola BL, Black DL, Brooks AN, Cheng C, Conesa A, Crosse EI, Eyras E, Guccione E, 2024. Steering research on mRNA splicing in cancer towards clinical translation. Nat Rev Cancer 24: 887–905. 10.1038/s41568-024-00750-2 39384951 PMC11698124

[GAD353988ARVC2] Bennett CF, Krainer AR, Cleveland DW. 2019. Antisense oligonucleotide therapies for neurodegenerative diseases. Annu Rev Neurosci 42: 385–406. 10.1146/annurev-neuro-070918-050501 31283897 PMC7427431

[GAD353988ARVC3] Ernst T, Chase AJ, Score J, Hidalgo-Curtis CE, Bryant C, Jones AV, Waghorn K, Zoi K, Ross FM, Reiter A, 2010. Inactivating mutations of the histone methyltransferase gene EZH2 in myeloid disorders. Nat Genet 42: 722–726. 10.1038/ng.621 20601953

[GAD353988ARVC4] Inoue D, Chew G-L, Liu B, Michel BC, Pangallo J, D'Avino AR, Hitchman T, North K, Lee SC-W, Bitner L, 2019. Spliceosomal disruption of the non-canonical BAF complex in cancer. Nature 574: 432–436. 10.1038/s41586-019-1646-9 31597964 PMC6858563

[GAD353988ARVC5] Islam MR, Nagar P, McNaughton N, Heeamoni SA, Hasan MM, Kandel S, Mukta IJ, Tsakiroglou P, Dalton WB, Abdel-Wahab O, 2026. Targeting *EZH2* oncogenic splicing: decoding the regulatory network and antisense correction. Genes Dev (this issue). 10.1101/gad.353628.126 PMC13474417 42547267

[GAD353988ARVC6] Kim E, Ilagan JO, Liang Y, Daubner GM, Lee SC-W, Ramakrishnan A, Li Y, Chung YR, Micol J-B, Murphy ME, 2015. SRSF2 mutations contribute to myelodysplasia by mutant-specific effects on Exon recognition. Cancer Cell 27: 617–630. 10.1016/j.ccell.2015.04.006 25965569 PMC4429920

[GAD353988ARVC7] Leclair NK, Brugiolo M, Urbanski L, Lawson SC, Thakar K, Yurieva M, George J, Hinson JT, Cheng A, Graveley BR, 2020. Poison Exon splicing regulates a coordinated network of SR protein expression during differentiation and tumorigenesis. Mol Cell 80: 648–665.e9. 10.1016/j.molcel.2020.10.019 33176162 PMC7680420

[GAD353988ARVC8] McGlincy NJ, Smith CWJ. 2008. Alternative splicing resulting in nonsense-mediated mRNA decay: what is the meaning of nonsense? Trends Biochem Sci 33: 385–393. 10.1016/j.tibs.2008.06.001 18621535

[GAD353988ARVC9] Rahman MA, Lin K-T, Bradley RK, Abdel-Wahab O, Krainer AR. 2020. Recurrent SRSF2 mutations in MDS affect both splicing and NMD. Genes Dev 34: 413–427. 10.1101/gad.332270.119 32001512 PMC7050488

[GAD353988ARVC10] Thomas JD, Polaski JT, Feng Q, De Neef EJ, Hoppe ER, McSharry MV, Pangallo J, Gabel AM, Belleville AE, Watson J, 2020. RNA isoform screens uncover the essentiality and tumor-suppressor activity of ultraconserved poison exons. Nat Genet 52: 84–94. 10.1038/s41588-019-0555-z 31911676 PMC6962552

